# Determinants of individual income in EU countries: implications for social policy targeting

**DOI:** 10.3389/fsoc.2023.1205094

**Published:** 2023-12-15

**Authors:** Irena Baláková, Jana Stávková, Petr Hudec

**Affiliations:** Department of Marketing and Trade, Faculty of Business and Economics, Mendel University in Brno, Brno, Czechia

**Keywords:** income inequality, individual income, Income Index, EU-SILC, income determinants

## Abstract

**Introduction:**

The introduction of the Income Index constructed by authors as well as the identification of demographic, socio-economic and occupation-related factors influencing the income of individuals in EU countries is the main contribution of the paper. The Income Index makes it possible to analyze data of individuals from all EU countries.

**Methods:**

The multiple hierarchical regression of EU-SILC microdata provides the factors that influence individuals’ income.

**Results:**

Outcomes show through which factors can be intervened in social policy settings to reduce income inequality. Factors significantly affecting the Income Index are the household composition, occupation sector (typically agriculture and accommodation and services are related to low incomes) and the degree of urbanization (rural areas with the lowest incomes of individuals).

**Discussion:**

Findings confirm ongoing discussions about the specific position of single parent households in the labour market and their need for social support.

## 1 Introduction

Household income is a crucial factor determining living conditions and satisfaction with the standard of living achieved (Yu et al., 2020). Household income reflects the socio-economic situation of the population of a given country (Bussolo et al., 2018). Boter (2020) explains that income depends on the life cycle, which is related to the living standard.

The European Union has common goals in the area of living standards, such as to promote the well-being of its citizens, combat social exclusion, reduce the number of people at risk of income poverty etc., (European Union, 2022). EU-SILC data, which provide information on the income situation and living conditions of households in the EU, can be used to assess the achievement of these objectives as well as for analyses providing data for decision making process of social policy makers. Many studies such as Jianu et al. (2021) and Soava et al. (2020) use the Gini coefficient as the indicator of income inequality in the context of income poverty. However, the Gini coefficient captures inequality in a limited way without detailed look at household income situation, hence there is a need for a multi-parameter model to more accurately explain income inequality (Blesch et al., 2022). Another way to compare rather global inequalities can be the analysis of purchasing power parities (PPP), which is an aggregate analysis suitable for developing policy measures aimed at reducing welfare disparities (Surinov and Luppov, 2021). Purchasing power parity analysis is appropriate when income inequalities are examined to identify consumption inequalities between several countries (Åberg Yngwe et al., 2005). However, it does not provide detailed data to identify the determinants of income inequality within a specific country. An indicator suitable for comparing the effects of various factors on the income situation of households across all EU member states simultaneously is notably absent from the EU’s uniform methodology. The authors of the paper have long been aware of the need to construct an indicator that would enable this comparison. The methodology of creating such an indicator is part of the paper.

This paper aims to identify the determinants of an individual’s income that have a decisive impact on their household income and thus on their living conditions. It is impossible to compare individuals’ incomes in absolute terms due to differences in average incomes and price levels across EU countries. These differences would make it impossible to observe the effects of the factors across EU countries. Therefore, another objective of the paper is to contribute to the search for a method allowing comparisons of effects of the determining factors on income between EU countries. For this purpose, the authors aim to create an income-based index called the “Income Index.” The final model containing the Income Index as the dependent variable is validated by robust analysis in selected countries representing cultural affinity zones in the EU. According to Usunier and Lee (2013), cultural affinity zones display similar characteristics for easily identifiable criteria such as language, religion, family life patterns, work relations and consumption patterns.

The paper is structured as follows. The following section reviews the literature concerning individual income and factors influencing income inequality. The next section introduces the data and methods used. The section called Results and Discussion provides the main model and the robustness analysis. The last section contains the conclusion and summary.

## 2 Literary review

Individuals’ income affects their welfare (Wolff, 2009). Income is a basic indicator of consumers’ living conditions (Stiglitz et al., 2009; Keeley, 2015) because the income enables consumers to meet their needs and influences consumption behavior (OECD, 2020). Consumers choose among a range of consumption possibilities subject to a given income constraint (Sloman and Wride, 2009). There are also other factors influencing consumers’ decision-making such as situational factors but the final decision is “determined by the wallet” (Achar et al., 2016). Burlacu (2016) also considers income to be the main source of consumption and savings, which are the determinants of the standard of living. The income and subsequent consumption shape consumers’ life satisfaction (Ambrey and Fleming, 2014). Atkinson and Marlier (2010) base their assessment of living conditions on income which includes earnings from employment, self-employment or retirement pensions and other social incomes.

### 2.1 Income inequality

However, individuals’ incomes vary and so do the consumption possibilities that shape their living conditions. The usefulness of household income for assessing inequalities, which further determine the different living conditions of households, is highlighted by Salverda et al. (2009). Some authors emphasise the need for inequality, such as Bussolo et al. (2018) who see the income inequality as a fundamental characteristic of a healthy society and a necessary consequence of a functioning economic system. According to Moller et al. (2009), income inequality is a function of economic development. On the other hand, some authors object that income inequality has a negative impact on the population, especially low-income households, and needs to be reduced or monitored and contained at an acceptable level (Greig et al., 2007; Salverda et al., 2009; Perkins et al., 2012; OECD, 2015). An appropriate assessment of income inequality is allowed by EU-SILC microdata (Atkinson and Marlier, 2010) and it is also possible to use EU-SILC in order to evaluate socioeconomic inequalities (Rubio Valverde et al., 2021). Similarly, Krell et al. (2017) state that extremely important for international social science research and policy advice on inequality. Carranza et al. (2022) use the Gini coefficient calculated from EU-SILC data to measure income inequality. However, they indicate that the Gini coefficient needs adjustments and they suggest that a more comprehensive inclusion of administrative income data should be a priority for European income statistics. Similarly, Hlasny and Verme (2018) point out the shortcomings of the Gini coefficient and recommend its reweighting.

Mogila et al. (2022) applied the EU-SILC dataset to search for factors affecting income inequality at the level of selected countries. The EU-SILC dataset has been used in studies not only in the income domain but also in research on living conditions, such as Hollederer and Wildner’s (2019) study on the link between income, health and education. Rubio Valverde et al. (2021) point to the social disabilities of the less educated. Income inequality in relation to health based on EU-SILC data has also been examined by Elstad (2016) and highlights the availability of health care in times of crisis.

The EU-SILC dataset can be used to determine the factors that shape household income. This was confirmed, for example, in a study by Soltes and Soltesova (2014), who used equivalized household income to determine the factors affecting it in a regression for the case of Slovakia. Dzuka et al. (2019) uses EU-SILC data and equivalized household income, but for a different purpose, namely to determine the impact of household income on well-being. Another example of a dataset used for household income analyses is the real-life datasets from banks by Kibekbaev and Duman (2016). Another option is specific databases targeting a particular area of economic activity such as the Situation Assessment Survey of Agricultural Households (SASAH) applied by Das and Srivastava (2021). However, there is no possibility of applying a uniform methodology for multiple countries in studies by Kibekbaev and Duman (2016) and Das and Srivastava (2021).

### 2.2 Factors affecting an individual’s income

Although income inequality can be greatly influenced by policy decisions (Chatterjee and Turnovsky, 2012; Coibion et al., 2017), factors relating to specific individuals and households are crucial (Wolff, 2009). Many studies have confirmed that the income situation of a household determined by the income situation of the individuals belonging to the household depends on the demographic and socio-economic characteristics (factors) of household members (Wolff, 2009). Corsi et al. (2016) showed that demographic characteristics, such as gender, determine income levels achieved by individuals, with women earning lower incomes than men. However, there raises a question of whether such income differences result from gender discrimination or whether men and women prefer careers in different sectors (with varying income levels). They explain that, for example, the service sector is typically female-dominated and generally offers lower wages or salaries. On the other hand, the information technology sector is perceived as a typically male-dominated higher-pay sector. Gender pay gaps in the labor markets are observed in EU countries (Castellano and Rocca, 2014). On average, women are paid 15% less than men in the EU (Boll and Lagemann, 2019). Kramer et al. (2016) explain that the gender pay gap leads to a situation where women do not have equal opportunities to achieve the same living conditions as men. Based on EU-SILC data, Mogila et al. (2022) conclude that public interventions should address gender discrimination. Navarro and Salverda (2019) found that men and women have different preferences when choosing jobs. These authors concluded that men tend to be more satisfied if they bring the highest earnings to the household and thus seek demanding jobs with higher pay. Most women, on the other hand, prefer jobs that allow them to spend more time with their families; hence the amount of income or salary is not the main deciding factor for them. This is consistent with Jost and Möser (2023) highlighting the heterogeneity of male and female preferences. In this context, Duvivier and Narcy (2015) point out the “motherhood penalty,” where women are often forced to interrupt their careers due to motherhood and subsequently fall behind men in their professional lives.

Another demographic factor, according to study by Corsi et al. (2016), is the age of the individuals. Income is expected to increase with age and experiences, but the situation may be reversed and, in the case of gender, there may be discrimination on the basis of age (Jyrkinen and Mckie, 2012; Vauclair et al., 2015). McGann et al. (2016) states that the ageism is differently perceived by men and women. The nature of ageism experienced by older women in administrative occupations appears like “lookism” that barely feature in the accounts of discrimination reported by men in managerial positions. On the other hand, Allgood (2020) highlights an age penalty at the start careers. Another critical factor that has previously been proven to affect individual’s income is the level of education, higher education of individuals is associated with higher income (Branyiczki, 2015; Bucevska, 2019). Tasseva (2021) states that higher education leads to higher wages, however, she argues that since the expansion of education in recent years has led to a larger increase in income in the middle and upper part of the distribution than in the lower part, income inequality has increased.

In addition to the highest educational attainment, Branyiczki (2015) discusses the influence of factors related to household members’ occupation and “work intensity” – in other words, how many hours are household members willing to devote to work. Vojtková and Šoltes (2018) confirm the influence of work intensity on personal income in the direction of the higher the work intensity, the higher the income. These authors explain work intensity as the capitalization on an individual’s work potential. Corsi et al. (2016) also verified the influence of economic activity and related influence of employment type (occupation and contract choice) on an individual’s income. Dafermos and Papatheodorou (2013) also agree with the effect of employment type on income.

Veneri and Murtin (2019) see economic activity as the primary determinant of the income level of individuals who collectively make up household income. These authors suggest adding another explanatory factor that is not as closely related to income, namely the individuals’ level of health. The association between income and health was verified by Gongora-Salazar et al. (2022). However, health does not have to be a determinant of income, but income can act as a determinant of health (Cui and Chang, 2021).

The individuals’ income is further influenced by the place of residence and degree of urbanization, where individuals in larger cities achieve higher incomes compared to individuals living in rural areas (Di Meglio et al., 2018). As Campbell (2021) explains, metropolitan residents experience a higher living costs compared to rural areas so they need higher income. On the other hand, Mogila et al. (2022) based on a regional analysis using EU-SILC data did not reveal substantial differences between urban and non-urban areas. The last frequently discussed determinant of household income is the household composition. According to Kis and Gábos (2016), bigger families who have lower levels of education often achieve low incomes. Di Meglio et al. (2018) points out that the most vulnerable households in terms of low incomes are single parent households.

Based on the literature review, the following hypotheses are established: H_0_ (1): The individual’s income is dependent on the gender (Kramer et al., 2016). H_0_ (2): The individual’s income is dependent on the age (Corsi et al., 2016). H_0_ (3): The individual’s income is dependent on the highest attained education (Tasseva, 2021). H_0_ (4): The individual’s income is dependent on the work intensity (Vojtková and Šoltes, 2018). H_0_ (5): The individual’s income is dependent on the place of residence (Di Meglio et al., 2018).

## 3 Materials and methods

The primary data source for the research is the EU-SILC survey (European Union - Statistics on Income and Living Conditions), specifically, EU-SILC 2021. The extensive EU-SILC microdata set provides detailed information on the income situations of households and individuals. The data also allow categorization of households’ and individuals’ specifics in terms of various demographic and socio-economic factors. The datasets include the Personal Weight variable which determines the number of individuals in the population, i.e., it allows the extrapolation of the EU-SILC data to the whole population of a given country. The EU-SILC survey is mandatorily carried out in all EU countries (Eurostat, 2021). The methodology for conducting the survey itself, as well as the methodology for processing the results, is drawn up by Eurostat.

The baseline variable for the presented research is the Individual’s income calculated as a sum of following variables: Employee cash income, Cash benefits or loses from self-employment, Pension from individual private pension plans, Unemployment benefits, Old-age benefits, Survivor’s benefits, Sickness benefits, Disability benefits and Education related allowances. The study had a total of 503,325 EU-SILC 2021 respondents from all EU countries in all economic activity categories aged 18 years and older.

Incomes of households from different countries cannot be compared in absolute terms. Therefore, a new variable that allows comparisons based on relative values is constructed and called the Income Index. The data from the individual EU countries are first analyzed separately. Subsequently, the Income Index is calculated for each dataset for each EU country. The Income Index is defined as the income percentile based on the Individual’s income variable (a sum of incomes explained above). The procedure for calculating the index is as follows:

1.All observations in datasets (all variables in all country EU-SILC datasets) are ordered from the lowest to the highest Individual’s income.

2.Then the cumulative share from the *Personal Weight* variable (*PW*) is calculated:

$$n_{j}=\frac{PW_{1}}{\sum PW};\frac{PW_{2}}{\sum PW};\ldots \frac{PW_{k}}{\sum PW}$$

The Personal Weight (PW) is part of the EU-SILC database and indicates the weight of a particular respondent within the total population.

3.Another auxiliary variable m is created as follows. The percentile is calculated based on the cumulative share.

$$m_{1}=n_{1};m_{2}=n_{2}+m_{1};\ldots m_{k}=n_{k}+m_{k-1}$$

$$\mathrm{Income}\;\mathrm{percentile}=m_{1}*100;\ldots m_{k}*100$$

Each individual in the dataset is thus assigned to a particular income percentile called the *Income Index*. The *Income Index* makes it possible to combine datasets from individual countries into one large set and perform regression analysis on this set, i.e., to include all EU countries with different income levels in one regression analysis. In the analysis, the *Income Index* acts as the dependent variable. The independent factors and their categories are summarized in the following Table 1. Dummy variables were created in order to apply nominal variables in the regression analysis. Reference categories are marked with an asterisk* in the Table 1.

**Table 1:** Independent factors in the regression model.

Gender (1 – man, 0 – woman*)
Age (18 years and older)
Education (1 – primary*, 2 – secondary, 3 – tertiary)
Economic status (1 – employees, 2 – unemployed, 3 – retired, 4 – unable to work, 5 – student, 6 – fulfilling domestic tasks, civilian service and other, 7 – entrepreneurs*)
Full-time working months (number of months working full-time)
Working hours per week (number of working hours per week)
Occupation (categories based on NACE Rev. 2: 1 – agriculture, forestry*, 2 – mining, energies, manufacturing, construction; 3 – wholesale, retail, transportation, storage; 4 – wholesale, retail, transportation, storage; 5 – information and communication; 6 – finance, insurance, real estate, administration; 7 – public administration, education, health)
Contract (1 – fixed, 0 – permanent*)
Supervisory responsibility (1 – yes, manager; 0 – no*)
Health (1 – very good; 2 – good, 3 – fair, 4 – bad, 5 – very bad*)
Household size (number of household members)
Household type (1 – one-person, 2 – single parent*, 3 – couple without children, 4 – couple with children, 5 – other)
Degree of urbanization (1 – city, 2 – town, suburbs, 3 – rural areas*)

Source: EU-SILC microdata (Eurostat, 2022), own processing in IBM SPSS Statistics.

Multiple linear regression analyses were based on the following general model:

$$y=\beta_{0}+\beta_{1}x_{1}+\beta_{2}x_{2}+\ldots +\beta_{m}x_{m}+\varepsilon$$

Where $x_{i}$ are independent (explanatory) variables, y is the dependent variable, $\beta_{i}$ are the unknown regression parameters, *m* stands for the number of the independent variables and ε represents the random file of the model (Greene, 2018). Variables of a non-ordinal nature enter the hierarchical regression analysis in the form of dummy variables. The reference categories(*) are shown in Table 1. The quality of the model in the OLS multiple regression analysis is evaluated by the coefficient of determination and ANOVA. The significance of the factors (independent variables) is verified by a t-test with the significance level of α = 0.05. When performing multiple linear regression, the authors employed the Enter method. The possible multicollinearity of the explanatory variables is verified by the VIF (Variance Inflation Factor) and Tolerance indicators. If there are no multicollinearity issues in the model, VIF values should be less than 10 and Tolerance values greater than 0.2 (Hebák et al., 2015). Statistical analyses were performed in using IBM SPSS Statistics 29 software.

## 4 Results

To identify the factors influencing the income of individuals in EU countries and determine the strength of the factors’ influence, regression analyzes were performed. Five representative EU countries were selected for regression analyses and verification of the application of the constructed Income Index. The Income Index was created by classifying individuals into an income percentile based on the sum of all incomes of an individual (income from employment, from business, social benefits, etc.). The independent variables in the regression models explain from 37 to 58% of the variance of the dependent variables (see the Appendix Tables A–E). The newly created Income Index is also affected by other factors not included in the EU-SILC survey. According to the value of *p* = 0.000 obtained by the F-test, all models are significant. Based on the VIF and Tolerance values, we can conclude that there is no multicollinearity in the models. The final models include only statistically significant variables or their categories in the case of nominal variables with a *t*-test value of *p* = 0.000.

The primary consideration for selection of country representatives was their division by zones of cultural affinity – based on Usunier and Lee’s (2013) distinction between groups of countries in Northern (Scandinavian), Central, Mediterranean and Anglo-Saxon Europe. The Benelux countries and Germany belong simultaneously to the Anglo-Saxon and Central European countries (Usunier and Lee, 2013). Finland has been chosen to represent Northern Europe, the Czech Republic to represent Central Europe, Italy to represent Mediterranean Europe, Ireland to represent Anglo-Saxon Europe. Romania is added to represent the EU countries with low economic performance. The countries selected for the robustness check also differ from each other in the year in which they joined the EU (European Parliament, 2022).

Table 2 provides a summary of the results of regression analyses carried out separately in selected countries. The summary allows a comparison of specific effects of independent variables on the dependent variable (*Income Index*) in different countries. The unstandardized coefficients show the direction of the dependence. All factors in the Table 2 are statistically significant and influence the *Income Index* except *Household size* in Ireland and *Household type (one-person)* in Ireland and in Romania (marked with “x”). The variable *Household type* has not been surveyed in Italy, therefore values are missing (“-”). Predictor values for which differences were found between countries with different impacts are shown in bold. The complete results of the regression analysis models for the five countries are available in the Appendix.

**Table 2:** Factors influencing the Income Index in different countries.

	Unstandardized coefficients B				
	FI	CZ	IT	IE	RO
(Constant)	−35.05	−63.25	−10.39	−14.77	−16.45
Gender (man)	4.86	12.62	4.81	5.22	7.64
Age	0.43	0.30	0.41	0.52	0.20
Education (secondary)	Ref.	28.32	8.70	2.30	12.22
Education (tertiary)	11.13	38.42	17.84	11.00	25.05
Economic status (employees)	28.69	16.36	8.24	22.92	32.35
Full-time working months	2.38	2.70	1.76	1.36	3.05
Working hours per week	0.22	0.06	0.14	0.39	0.12
Occupation (mining, energies, manufacturing, construction)	8.25	0.63	7.77	9.84	2.93
Occupation (wholesale, retail, transportation, storage)	2.65	1.09	7.35	6.18	2.33
Occupation (accommodation, food service, arts)	−2.77	−7.53	−1.72	**4.03**	−1.10
Occupation (information and communication)	11.76	12.20	11.91	10.86	10.20
Occupation (finance, insurance, real estate, administration)	4.65	7.34	8.55	8.66	8.18
Occupation (public administration, education, health)	3.41	7.29	8.44	12.33	11.46
Contract (fixed = not permanent)	−7.37	−5.63	−9.64	−6.31	−0.82
Supervisory responsibility	5.86	10.79	5.18	6.94	6.31
Health (very good)	13.97	21.53	6.18	**−14.19**	**−18.87**
Health (good)	11.89	19.14	6.03	**−15.28**	**−20.96**
Health (fair)	11.55	15.66	4.79	**−15.57**	**−23.49**
Health (bad)	17.94	11.30	3.32	**−23.97**	**−27.53**
Household size	−0.37	**1.19**	**0.55**	x	−0.84
Household type (one-person)	**−3.25**	3.62	–	x	x
Household type (couple without children)	**−2.02**	0.80	–	0.15	0.77
Household type (couple with children)	1.43	0.31	–	1.08	3.07
Household type (other)	−3.31	−1.15	–	−2.41	**2.02**
Degree of urbanization (city)	2.23	5.31	**−0.38**	3.20	5.61
Degree of urbanization (town, suburbs)	0.69	1.55	**−0.04**	4.21	0.92

Source: EU-SILC microdata (Eurostat, 2022), own processing in IBM SPSS Statistics.Predictor values for which differences were found between countries with different impacts are shown in bold.

The results (Table 2) show that some variables, due to their nominal nature, had to be applied in the model as dummy variables (*Gender, Education, Economic status* and others that are not interval such as *Age* or *Working hours per week*) with interpretation in relation to the reference category.

The calculation from EU-wide data gave the following results. As for demographic factors, the negative effect of *Gender* on the *Income Index* in all countries shows that women have lower incomes than men in the labor market and in terms of social incomes as well. The first hypothesis of gender and income dependence is confirmed. Men achieve 5.7 points more than women on the income percentile scale. The influence of the Age on the income is statistically significant and the null hypothesis n.2 of age and income dependence is confirmed. The *Income Index* increases with increasing *Age*.

There is a statistically significant dependence for the highest attained education, which confirms the third hypothesis of dependence of these variables. The reference category is primary education. Individuals with a university (tertiary) education have higher incomes than those with a primary education; the situation is similar for those with a secondary education. The Finnish data does not include a primary education category, so secondary education was set as the reference variable. However, it is confirmed that tertiary education leads to a higher Income Index compared secondary education.

*Economic status* with the reference variable self-employed shows that employees earn higher incomes than small entrepreneurs. For the number of months worked in the main economic activity as well as hours worked per week, the expected positive relationship is confirmed. The null hypothesis of work intensity and income dependence is confirmed. Individuals who do not change employment frequently seem to have higher incomes.

When investigating income differences across sectors (*Occupation*), the agriculture and forestry sector was taken as the reference category. Statistically significant differences are found in the average income in the information and communication sector where individuals earn higher incomes compared to those working in the agriculture and forestry. A similar trend is found in the other sectors with the exception of accommodation and food services, where individuals achieve lower incomes compared to agriculture. However, this is not only true in Ireland where individuals working in the accommodation area earn more than individuals active in the agriculture. As for the type of employment *contract*, permanent contracts mean higher earnings compared to fixed contract, as well as if the individual holds a managerial supervisory position in all countries.

The greatest differences among countries are observed in the variable related to the *Health* of the individual. Better health scores have not been confirmed to lead to higher income percentiles in Ireland and Romania. The source of the differences may also be in the fact that this is a subjective assessment of health and different nations take different optimistic or pessimistic approaches to health perceptions.

The last variables in the model relate to the household in which the individual lives. It is possible to observe differences between countries. In terms of *Household type*, most types of households in the observed countries are stronger in income compared to single parent households with children. The only exception is Finland.

According to the *Degree of urbanization*, incomes are higher in cities and also towns and suburbs compared to rural areas. The last hypothesis of residence and income dependence is confirmed. Italy is an exception in this variable. Individual incomes in cities and towns appear to be slightly low compared to rural areas.

## 5 Discussion and conclusions

The main contribution of this paper is the possibility of comparing the impact of factors on the income of individuals across the European Union through the construction and application of the *Income Index*. Previous studies such as Jianu et al. (2021) and Soava et al. (2020) have used, for example, the Gini coefficient to compare income inequalities across countries, but it cannot be used to identify the factors that affect an individual’s income itself because it is the overall macroeconomic indicator and do not represent the level of individuals. Similarly, Hlasny and Verme (2018) and Carranza et al. (2022) discuss income inequality and the Gini coefficient with possible adjustments, but do not address in detail what factors cause income inequality. They also do not consider the possibility of an income indicator that could become part of the methodology for European statistics and be applicable to more countries. A weakness of the equivalized disposable income used by Dzuka et al. (2019) and Soltes and Soltesova (2014) is that it reflects the income of the entire household, not of a particular individual, which is shaped by the demographic and socioeconomic factors that relate to the individual. Therefore, in this study, the basis for the Income Index is the sum of incomes that is related only to a particular individual. Carranza et al. (2022) mention that data collection across the EU and a harmonized methodology is a challenge. One of the contributions of this study is the design of the Income Index that can be used as an income indicator to compare the income situation of more countries.

The EU’s long-term goal is to reduce the poverty rate in the Member States (European Union, 2022), which according to the current methodology used to measure the poverty rate also means reducing income inequalities, it is necessary to know which factors to focus on. This paper provides clues to finding a solution to this problem. It is possible to confirm Atkinson and Marlier (2010) that EU-SILC microdata enable comparison of income inequalities across EU countries. A weakness, also mentioned by Čižmešija et al. (2018), is the inability of EU-SILC to respond quickly to turbulent changes in the social and economic dimensions of individuals’ well-being (e.g., changes caused by the Covid-19 pandemic).

The impact of different factors on income and resulting poverty Halleröd and Larsson (2008) should be considered when preparing social policy instruments. Countries that have achieved the most significant reductions in poverty rates after payment of social transfers are Ireland and Finland. Ireland achieved a 17.9% reduction of poverty and Finland a 13.6% reduction of poverty in 2019 (Eurostat, 2022) through an appropriate combination of social policy instruments. Ireland can be seen as a model country.

The effect of fundamental demographic factors (*Gender*, *Age*, etc.) and socio-economic factors (*Education*, *Household size and Household type*) on income was confirmed as expected. Measures reducing the gender pay gap are one of the EU’s priorities. Member States committed themselves in 2003 to follow the European Employment Strategy (EES), which aimed to halve the gender pay gap by 2010 (Rubery et al., 2005). The income equalization between the genders has not been achieved to this day. According to the European Commission (2021), the gender pay gap in the EU is 13%. Toczek et al. (2021) point out that the income developments of men rise more sharply than those of women over time.

It is confirmed that individuals at older ages have higher incomes. However, there are countries in which this dependence stagnates from a certain age (Jyrkinen and Mckie, 2012). Income discrimination does not only affect gender and age categories but it also affects the entire labor market (Cavalcanti and Tavares, 2016). University educated individuals achieve a higher income. The highest attained education is linked to the type of position and responsibilities in the labor market, which is likely to shape income inequalities, not discrimination. The education level is one of the main determinants of economic income alongside age and gender (Mamani et al., 2022). This finding suggests the need for social support for single-parent households because they achieve the lowest incomes.

When examining *Economic status*, it was found that employees achieve the higher *Income index* than the self-employed in the labor market, which is consistent with Bradley et al. (2015). Since factors determining the time spent at work (*Full-time working months and Working hours per week*) have also been found to be significant, it is advisable to support those who cannot be 100% economically active. That’s quite typical for single-parent who look after children and work part time. Another variable significantly affecting an individual’s income is the sector (*Occupation*) in which the individual works, as some sectors, such as agriculture and forestry and also accommodation and services, are characterized by lower remuneration which is in agreement with Hedija (2014). Antošová et al. (2021) found out that the agricultural household income does not reach the average household income in any of the EU countries. Similarly, Herman (2016) drew attention to the lower personal incomes in the agricultural sector, specifically to the relation between working poverty and the employment in agriculture.

The model shows that permanent *contracts* compared to fixed contracts are related to higher incomes. The forms of employment contracts should be regulated and employers should be encouraged to provide permanent contracts that ensure, in addition to higher incomes, a stable basis for satisfactory living conditions for households. Another verified finding is that the *Income Index* increases with *Supervisor responsibility*. There is a possibility that some examined factors influence others, for example, *Education* may influence *Occupation* or *Supervisor responsibility*, which could be an area for further research.

Regarding the *Degree of urbanization*, it was revealed that individuals living in cities and towns achieve higher *Income percentile* compared to individuals living in rural areas which is in accordance with Campbell (2021). The last considered influence was subjective perceived *Health*, where individuals with better health were more likely to achieve higher incomes. This agrees with Hollederer and Wildner (2019), who report that the probability of better health increased with increasing disposable income. However, there were found differences in *Heath* variable in the robustness analysis. Borisova (2019) also stated that the subjective health in Central and Eastern European countries differ and is influenced by a mix of determinants.

The regression analysis results provide factors influencing income of individual. Actions through these revealed factors can be used in setting social policy and employment policy in the EU in the area of reducing household income poverty. By knowing the factors influencing an individual’s income, social assistance can be precisely targeted to income vulnerable individuals in need. It is possible to act with social benefits in the context of household composition and to support vulnerable groups (single parent households with children). In terms of occupation, some sectors such as agriculture and forestry and accommodation and services should be supported. It is also appropriate to support income-poor regions, i.e., rural areas. Permanent contracts have been confirmed to lead to higher incomes.

The issue that could be the area for further research is the inclusion of *Health* in the model, for which the direction of influence was different in two countries. The subjective assessment of the *Health* represents a limitation of this study. Another limitation may be the absence of additional variables in the model such as the income situation of the household partner. The time delay in the availability of EU-SILC data as discussed by Čižmešija et al. (2018) could be the research limitation as well.

## Data availability statement

The data analyzed in this study is subject to the following licenses/restrictions: The study uses microeconomic data from the EU-SILC survey (European Union Statistics on Income and Living Conditions). Data are available to research institutions on request. Requests to access these datasets should be directed to https://ec.europa.eu/eurostat/web/microdata/european-union-statistics-on-income-and-living-conditions.

## Author contributions

IB: Writing – original draft, review & editing, Conceptualization, Methodology, Data curation, Project administration. JS: Writing – original draft, Conceptualization, Methodology. PH: Writing – review & editing, Formal Analysis.

## Funding

Supported by the Mendel University in Brno, Czech Republic, Project No. IGA-PEF-TP-23-012.
