# Supplementary material for: Determinants of individual income in EU countries: implications for social policy targeting
Source: Front Sociol. 2023 Dec 15;8:1205094. doi: 10.3389/fsoc.2023.1205094 (PMC10754958; doi:10.3389/fsoc.2023.1205094)
Supplement: Supplementary file 1 [file Table_1.docx]

**Appendix**

Tab. A Factors influencing the EU Income Index (regression analysis results) in **Finland**

| Variables | Unstandardized Coefficients | | Standardized Coefficients | t |
| --- | --- | --- | --- | --- |
|  | B | Std. Error | Beta |  |
| (Constant) | -35.05*** | 0.565 |  | -61.985 |
| Gender (man) | 4.86*** | 0.030 | 0.113 | 161.729 |
| Age | 0.43*** | 0.001 | 0.245 | 364.526 |
| Education (tertiary) | 11.13*** | 0.030 | 0.260 | 377.169 |
| Economic status (employees) | 28.69*** | 0.290 | 0.062 | 99.094 |
| Full-time working months | 2.38*** | 0.005 | 0.393 | 454.954 |
| Working hours per week | 0.22*** | 0.003 | 0.072 | 88.151 |
| Occupation (mining, energies, manufacturing, construction) | 8.25*** | 0.142 | 0.158 | 58.183 |
| Occupation (wholesale, retail, transportation, storage) | 2.65*** | 0.143 | 0.046 | 18.551 |
| Occupation (accommodation, food service, arts) | -2.77*** | 0.148 | -0.036 | -18.802 |
| Occupation (information and communication) | 11.76*** | 0.152 | 0.123 | 77.415 |
| Occupation (finance, insurance, real estate, administration) | 4.65*** | 0.144 | 0.076 | 32.209 |
| Occupation (public administration, education, health) | 3.41*** | 0.143 | 0.074 | 23.888 |
| Contract (fixed = not permanent) | -7.37*** | 0.044 | -0.118 | -169.231 |
| Supervisory responsibility | 5.86*** | 0.030 | 0.128 | 195.269 |
| Health (very good) | 13.97*** | 0.452 | 0.288 | 30.872 |
| Health (good) | 11.89*** | 0.452 | 0.276 | 26.306 |
| Health (fair) | 11.55*** | 0.453 | 0.196 | 25.497 |
| Health (bad) | 17.94*** | 0.464 | 0.105 | 38.656 |
| Household size | -0.37*** | 0.024 | -0.022 | -15.307 |
| Household type (one-person) | -3.25*** | 0.064 | -0.074 | -50.494 |
| Household type (couple without children) | -2.02*** | 0.057 | -0.042 | -35.631 |
| Household type (couple with children) | 1.43*** | 0.065 | 0.030 | 21.867 |
| Household type (other) | -3.31*** | 0.207 | -0.011 | -16.011 |
| Degree of urbanization (city) | 2.23*** | 0.037 | 0.052 | 61.126 |
| Degree of urbanization (town, suburbs) | 0.69*** | 0.038 | 0.015 | 18.221 |

Dependent Variable: Income index. R = 0.76. R^2^ = 0.58.

Source: EU-SILC microdata (Eurostat, 2022), own processing in IBM SPSS Statistics

Tab. B Factors influencing the EU Income Index (regression analysis results) in the **Czech Republic**

| Variables | Unstandardized Coefficients | | Standardized Coefficients | t |
| --- | --- | --- | --- | --- |
|  | B | Std. Error | Beta |  |
| (Constant) | -63.25 | 0.714 |  | -88.565 |
| Gender (man) | 12.62 | 0.019 | 0.277 | 670.053 |
| Age | 0.30 | 0.001 | 0.147 | 341.751 |
| Education (secondary) | 28.32 | 0,658 | 0.555 | 43,041 |
| Education (tertiary) | 38.42 | 0.658 | 0.753 | 58.357 |
| Economic status (employees) | 16.36 | 0.168 | 0.037 | 97.408 |
| Full-time working months | 2.70 | 0.004 | 0.318 | 691.518 |
| Working hours per week | 0.06 | 0.002 | 0.014 | 30.274 |
| Occupation (mining, energies, manufacturing, construction) | 0.63 | 0.060 | 0.013 | 10.472 |
| Occupation (wholesale, retail, transportation, storage) | 1.09 | 0.062 | 0.018 | 17.426 |
| Occupation (accommodation, food service, arts) | -7.53 | 0.071 | -0.072 | -106.589 |
| Occupation (information and communication) | 12.20 | 0.077 | 0.096 | 158.803 |
| Occupation (finance, insurance, real estate, administration) | 7.34 | 0.067 | 0.089 | 109.821 |
| Occupation (public administration, education, health) | 7.29 | 0.062 | 0.141 | 117.782 |
| Contract (fixed = not permanent) | -5.63 | 0.033 | -0.068 | -169.197 |
| Supervisory responsibility | 10.79 | 0.024 | .0182 | 458.421 |
| Health (very good) | 21.53 | 0.192 | 0.445 | 112.335 |
| Health (good) | 19.14 | 0.191 | 0.421 | 99.985 |
| Health (fair) | 15.66 | 0.193 | 0.238 | 81.336 |
| Health (bad) | 11.30 | 0.202 | 0.066 | 56.001 |
| Household size | 1.19 | 0.014 | 0.061 | 81.901 |
| Household type (one-person) | 3.62 | 0.043 | 0.051 | 83.719 |
| Household type (couple without children) | 0.80 | 0.035 | 0.014 | 23.072 |
| Household type (couple with children) | 0.31 | 0.036 | -0.007 | -8.639 |
| Household type (other) | -1.15 | 0.058 | -0.009 | -19.719 |
| Degree of urbanization (city) | 5.31 | 0.023 | 0.108 | 234.674 |
| Degree of urbanization (town, suburbs) | 1.55 | 0.021 | 0.032 | 73.321 |

Dependent Variable: Income index. R = 0.66. R^2^ = 0.43.

Source: EU-SILC microdata (Eurostat, 2022), own processing in IBM SPSS Statistics

Tab. C Factors influencing the EU Income Index (regression analysis results) in **Italy**

| Variables | Unstandardized Coefficients | | Standardized Coefficients | t |
| --- | --- | --- | --- | --- |
|  | B | Std. Error | Beta |  |
| (Constant) | -10.39*** | 0.088 |  | -117.972 |
| Gender (man) | 4.81*** | 0.009 | 0.106 | 561.286 |
| Age | 0.41*** | 0.000 | 0.211 | 1122.955 |
| Education (secondary) | 8.70*** | 0.023 | 0.171 | 386.064 |
| Education (tertiary) | 17.84*** | 0.024 | 0.337 | 741.052 |
| Economic status (employees) | 8.24*** | 0.023 | 0.062 | 364.334 |
| Full-time working months | 1.76*** | 0.001 | 0.384 | 1763.883 |
| Working hours per week | 0.14*** | 0.001 | 0.051 | 234.334 |
| Occupation (mining, energies, manufacturing, construction) | 7.77*** | 0.022 | 0.159 | 346.948 |
| Occupation (wholesale, retail, transportation, storage) | 7.35*** | 0.023 | 0.122 | 315.916 |
| Occupation (accommodation, food service, arts) | -1.72*** | 0.024 | -0.024 | -71.624 |
| Occupation (information and communication) | 11.91*** | 0.031 | 0.09 | 388.262 |
| Occupation (finance, insurance, real estate, administration) | 8.55*** | 0.024 | 0.118 | 351.333 |
| Occupation (public administration, education, health) | 8.44*** | 0.023 | 0.159 | 361.988 |
| Contract (fixed = not permanent) | -9.64*** | 0.011 | -0.162 | -875.288 |
| Supervisory responsibility | 5.18*** | 0.009 | 0.101 | 591.09 |
| Health (very good) | 6.18*** | 0.075 | 0.118 | 82.917 |
| Health (good) | 6.03*** | 0.074 | 0.13 | 81.219 |
| Health (fair) | 4.79*** | 0.075 | 0.067 | 64.034 |
| Health (bad) | 3.32*** | 0.079 | 0.02 | 42.165 |
| Household size | 0.55*** | 0.003 | 0.03 | 178.197 |
| Degree of urbanization (city) | -0.38*** | 0.011 | -0.008 | -34.091 |
| Degree of urbanization (town, suburbs) | -0.04*** | 0.011 | -0.001 | -3.278 |

Dependent Variable: Income index. R = 0.71. R^2^ = 0.50.

Source: EU-SILC microdata (Eurostat, 2022), own processing in IBM SPSS Statistics

Tab. D Factors influencing the EU Income Index (regression analysis results) in **Ireland**

| Variables | Unstandardized Coefficients | | Standardized Coefficients | t |
| --- | --- | --- | --- | --- |
|  | B | Std. Error | Beta |  |
| (Constant) | -14.77 | 0.689 |  | -21.44 |
| Gender (man) | 5.22 | 0.027 | 0.119 | 193.755 |
| Age | 0.52 | 0.001 | 0.273 | 453.905 |
| Education (secondary) | 2.30 | 0.094 | 0.051 | 24.524 |
| Education (tertiary) | 11.00 | 0.095 | 0.247 | 115.519 |
| Economic status (employees) | 22.92 | 0.495 | 0.026 | 46.256 |
| Full-time working months | 1.36 | 0.004 | 0.285 | 359.175 |
| Working hours per week | 0.39 | 0.002 | 0.171 | 211.697 |
| Occupation (mining, energies, manufacturing, construction) | 9.84 | 0.154 | 0.179 | 63.912 |
| Occupation (wholesale, retail, transportation, storage) | 6.18 | 0.155 | 0.1 | 39.833 |
| Occupation (accommodation, food service, arts) | 4.03 | 0.158 | 0.049 | 25.489 |
| Occupation (information and communication) | 10.86 | 0.159 | 0.124 | 68.099 |
| Occupation (finance, insurance, real estate, administration) | 8.66 | 0.155 | 0.15 | 55.899 |
| Occupation (public administration, education, health) | 12.33 | 0.154 | 0.265 | 80.062 |
| Contract (fixed = not permanent) | -6.31 | 0.055 | -0.066 | -115.72 |
| Supervisory responsibility | 6.94 | 0.027 | 0.151 | 254.336 |
| Health (very good) | -14.19 | 0.449 | -0.322 | -31.627 |
| Health (good) | -15.28 | 0.449 | -0.336 | -34.058 |
| Health (fair) | -15.57 | 0.45 | -0.183 | -34.587 |
| Health (bad) | -23.97 | 0.46 | -0.12 | -52.116 |
| Household type (couple without children) | 0.15 | 0.042 | 0.002 | 3.51 |
| Household type (couple with children) | 1.08 | 0.033 | 0.025 | 32.68 |
| Household type (other) | -2.41 | 0.047 | -0.034 | -51.227 |
| Degree of urbanization (city) | 3.20 | 0.03 | 0.071 | 107.807 |
| Degree of urbanization (town, suburbs) | 4.21 | 0.032 | 0.086 | 133.089 |

Dependent Variable: Income index. R = 0.70. R^2^ = 0.49.

Source: EU-SILC microdata (Eurostat, 2022), own processing in IBM SPSS Statistics

Tab. E Factors influencing the EU Income Index (regression analysis results) in **Romania**

| Variables | Unstandardized Coefficients | | Standardized Coefficients | t |
| --- | --- | --- | --- | --- |
|  | B | Std. Error | Beta |  |
| (Constant) | -16.45*** | 0.452 |  | -36.386 |
| Gender (man) | 7.64*** | 0.013 | 0.197 | 605.009 |
| Age | 0.20*** | 0.001 | 0.107 | 309.44 |
| Education (secondary) | 12.22*** | 0.084 | 0.29 | 145.834 |
| Education (tertiary) | 25.05*** | 0.085 | 0.591 | 294.04 |
| Economic status (employees) | 32.35*** | 0.16 | 0.063 | 202.684 |
| Full-time working months | 3.05*** | 0.004 | 0.226 | 717.604 |
| Working hours per week | 0.12*** | 0.002 | 0.019 | 60.454 |
| Occupation (mining, energies, manufacturing, construction) | 2.93*** | 0.034 | 0.074 | 86.44 |
| Occupation (wholesale, retail, transportation, storage) | 2.33*** | 0.035 | 0.052 | 66.731 |
| Occupation (accommodation, food service, arts) | -1.10*** | 0.041 | -0.013 | -26.684 |
| Occupation (information and communication) | 10.20*** | 0.046 | 0.1 | 219.462 |
| Occupation (finance, insurance, real estate, administration) | 8.18*** | 0.04 | 0.116 | 204.713 |
| Occupation (public administration, education, health) | 11.46*** | 0.036 | 0.224 | 313.84 |
| Contract (fixed = not permanent) | -0.82*** | 0.025 | -0.01 | -32.987 |
| Supervisory responsibility | 6.31*** | 0.023 | 0.089 | 272.235 |
| Health (very good) | -18.87*** | 0.401 | -0.487 | -47.05 |
| Health (good) | -20.96*** | 0.401 | -0.545 | -52.251 |
| Health (fair) | -23.49*** | 0.402 | -0.283 | -58.47 |
| Health (bad) | -27.53*** | 0.442 | -0.046 | -62.349 |
| Household size | -0.84*** | 0.006 | -0.065 | -133.701 |
| Household type (couple without children) | 0.77*** | 0.021 | 0.014 | 35.978 |
| Household type (couple with children) | 3.07*** | 0.021 | 0.079 | 147.271 |
| Household type (other) | 2.02*** | 0.032 | 0.033 | 62.745 |
| Degree of urbanization (city) | 5.61*** | 0.016 | 0.143 | 348.275 |
| Degree of urbanization (town, suburbs) | 0.92*** | 0.016 | 0.022 | 57.697 |

Dependent Variable: Income index. R = 0.61. R^2^ = 0.37.

Source: EU-SILC microdata (Eurostat, 2022), own processing in IBM SPSS Statistics
